# Inner ear deficits complicate interpretation of depression-like behaviors in *Gpr156* mutant mice

**DOI:** 10.1073/pnas.2609064123

**Published:** 2026-08-24

**Authors:** Basile Tarchini, Ruth Anne Eatock, Katie S. Kindt, Cesare Orlandi, Kathleen E. Cullen

**Affiliations:** ^a^ The Jackson Laboratory, Bar Harbor, ME 04609; ^b^ Department of Neurobiology, University of Chicago, Chicago, IL 60637; ^c^ Section on Sensory Cell Development and Function, National Institute on Deafness and Other Communication Disorders, NIH, Bethesda, MA 20892; ^d^ Department of Pharmacology and Physiology, University of Rochester Medical Center, Rochester, NY 14642; ^e^ Department of Biomedical Engineering, Johns Hopkins University, Baltimore, MD 21205

The article by Miller et al., “*A rare variant in GPR156 associated with depression in a Mennonite pedigree causes habenula hyperactivity and stress sensitivity in mice*” (1), proposes an interesting link between GPR156 and depression-related phenotypes. However, well-established prior results demonstrating auditory and vestibular roles of GPR156 in mice are directly relevant to the interpretation of the behavioral assays reported in this study but were not considered by the authors.

Importantly, the mouse models examined by Miller et al. are either homozygous for knockout of *Gpr156* or homozygous for the depression-associated E533D allele, whereas the human subjects in the study are all heterozygous carriers of E533D. This difference in genetic context is critical given the known inner ear abnormalities due to recessive loss of function of *Gpr156.*

The knockout strain is presented as newly generated but corresponds to a model generated by the KOMP consortium based on a Velocigene design (RRID:MMRRC_047937-UCD). This strain has been available at the MMRRC repository and is the same strain we used for inner ear studies. The strain used is consequential for interpreting the behavioral data in Miller et al.

First, defective swimming behavior (figure 5) is interpreted as depression-like behavior. However, knockout mice carrying the same *Gpr156* null allele are known to exhibit severe vestibular dysfunction that impairs their ability to maintain head orientation and stability in water and also impairs the vestibulo-ocular reflex (2, 3). In this context, erratic swimming behavior in both knockout and E533D homozygous mice could arise from vestibular impairment rather than an altered affective state. Similarly, interpretation of the acoustic startle response (*SI Appendix*, figure S9) is problematic, as the knockout strain is known to exhibit hearing loss associated with abnormal hair cell orientation (4). Notably, heterozygous mice of these strains do not exhibit hearing loss, vestibular problems, or abnormal behavioral features, consistent with the recessive nature of these deficits. The omission of these well-established results raises the question of whether other behavioral phenotypes in the Miller study may reflect sensory deficits rather than depression-related behaviors.

Since our original discovery of *Gpr156* as a gene important for auditory function in mice (4), multiple damaging variants of GPR156 have been identified and associated with congenital hearing loss in humans (5–7). These studies were not considered by Miller et al. and further validate the relevance of GPR156 to inner ear function, reinforcing the significance of the sensory phenotypes to the behavioral assays in mice.

Finally, we have generated a hair cell-specific conditional *Gpr156* knockout strain and demonstrated that hearing and vestibular deficits arise specifically from misoriented sensory hair cells in the inner ear (8). Together with prior work, these findings indicate that inner ear dysfunction complicates the interpretation of several behavioral assays of homozygous mouse models used by Miller et al. We suggest that these sensory phenotypes should be carefully considered when evaluating the behavioral effects attributed to *Gpr156* mutations.

## References

[1] B. R. Miller , A rare variant in GPR156 associated with depression in a Mennonite pedigree causes habenula hyperactivity and stress sensitivity in mice. Proc. Natl. Acad. Sci. U.S.A. **122**, e2404754122 (2025). 40228124 10.1073/pnas.2404754122 PMC12037005

[2] K. Ono , Contributions of mirror-image hair cell orientation to mouse otolith organ and zebrafish neuromast function. Elife **13**, RP97674 (2024). 39531034 10.7554/eLife.97674 PMC11556791

[3] N. C. Hughes, D. C. Roberts, B. Tarchini, K. E. Cullen, Instrumented swim test for quantifying motor impairment in rodents. Sci. Rep. **14**, 29270 (2024). 39587238 10.1038/s41598-024-80344-y PMC11589839

[4] K. S. Kindt , EMX2-GPR156-Galphai reverses hair cell orientation in mechanosensory epithelia. Nat. Commun. **12**, 2861 (2021). 34001891 10.1038/s41467-021-22997-1 PMC8129141

[5] D. Greene , Genetic association analysis of 77,539 genomes reveals rare disease etiologies. Nat. Med. **29**, 679–688 (2023). 36928819 10.1038/s41591-023-02211-z PMC10033407

[6] M. Ramzan , Novel GPR156 variants confirm its role in moderate sensorineural hearing loss. Sci. Rep. **13**, 17010 (2023). 37814107 10.1038/s41598-023-44259-4 PMC10562426

[7] M. M. Aslam , A novel homozygous loss-of-function variant in GPR156 delineates non-syndromic hearing loss. Biochem. Genet. **64**, 89–95 (2025). 39760840 10.1007/s10528-024-11019-6

[8] A. Jarysta, B. M. Verdone, E. I. Hartig, K. E. Cullen, B. Tarchini, GPR156 is required in sensory hair cells for proper auditory and vestibular function. Sci. Rep. **16**, 4276 (2026). 41547998 10.1038/s41598-025-34476-4 PMC12859106

